# A Mixed Methods Pilot Study to Evaluate User Engagement with *MedMicroMaps*: A Novel Interactive *E*-learning Tool for Medical Microbiology

**DOI:** 10.1007/s40670-024-02047-3

**Published:** 2024-05-01

**Authors:** Jane Harrington

**Affiliations:** Montana College of Osteopathic Medicine, Rocky Vista University, Billings, MT USA

**Keywords:** Infectious diseases, Microbiology, Method of loci, Spatial memory, Mindmap, *E*-learning, Virtual reality, Augmented reality, Extended reality, Artificial intelligence, Machine learning, Metaverse

## Abstract

Educators are witnessing the unfolding of the era of artificial intelligence, raising the question of how to transfer the benefits of yesterday’s pedagogy to the future of education. An interactive digital mind map of infectious diseases was developed for second-year medical students (*n* = 865). Analysis of user engagement showed global distribution with 498 QR scans on a single day. Student responses (*n* = 79, 9.1% response rate) indicated positive feedback on the resources of Extremely Satisfied (range 65–75%, *n* = 59–51). The findings of the study support further expansion of MedMicroMaps to cross-platform interfaces with adaptations for diverse audiences within allied health fields.

**Supplementary Information:**

The online version contains supplementary material available at 10.1007/s40670-024-02047-3.

## Background

The transformation in preclinical medical education from predominantly didactic in-person lectures to reliance on *e*-learning modalities became apparent in 2020 in context of the COVID-19 pandemic [1]. Once students returned to on-campus learning, medical educators faced the quandary of how to best leverage an effective balance of digital content with traditional oral content delivery to meet the needs of a younger generation of learners. Ranges of learning preferences for diverse populations of medical student necessitate development of *e*-learning modalities that allow flexibility of student-led inquiry which is adaptable to approaching evidence-based medicine with both basic sciences and clinical reasoning [2]. Visual learning in *e*-learning study guides with interactive elements confers a benefit over strictly text course content for non-traditional learners including non-native English learners and neurodiverse individuals [3]. Mind maps and the Method of Loci *aka* Memory Palace are pedagogical strategies that use spatial positioning, color-coding, and repeated symbolism to create pattern associations for more efficient memorization and are readily adaptable to *e*-learning modalities including web-based resources and immersive technologies [4, 5]. A comprehensive interactive mind map for infectious diseases *aka* MedMicroMaps was developed in response to global virtual learning during the COVID-19 pandemic to guide medical students through differential diagnoses with options to approach case-based scenarios with clinical, epidemiological, and biological organization algorithms.

## Activity

**Fig. 1:**
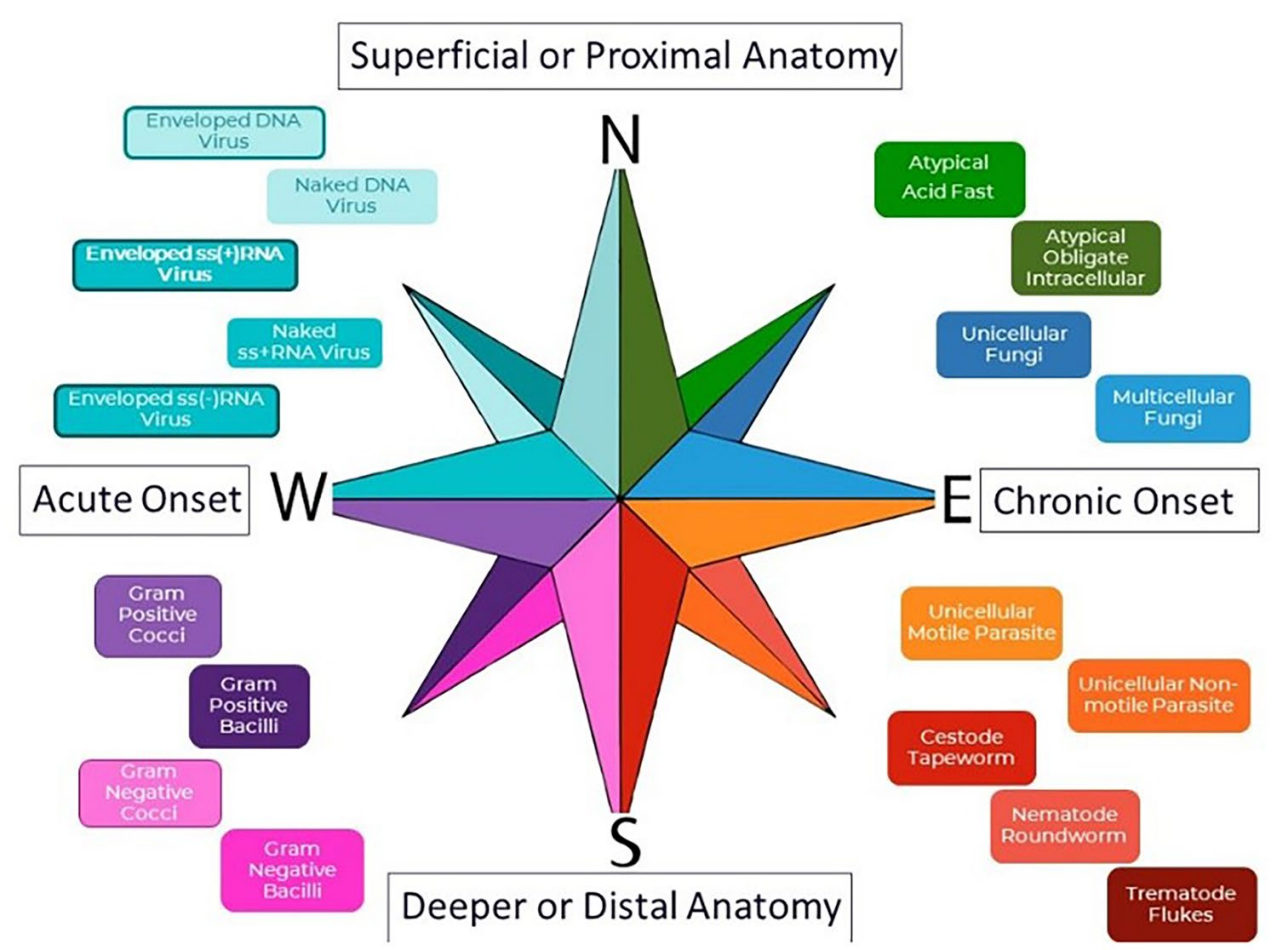
MedMicroMaps compass. An interactive guide of infectious diseases on PowerPoint platform was developed for preclinical medical students, using the principles of mind maps and Method of Loci to create a consistent color-coding and spatial patterns arranged on a compass with cardinal directions. North-to-south corresponds to anatomical location and west-to-east corresponds onset of clinical presentation. Color-coded microbes are arranged north-to-south based on smaller to larger, with subset patterns of classification (viruses: DNA on top and RNA on bottom, with border to indicate enveloped and no border for non-enveloped)

The initial impetus for developing the MedMicroMaps system was to create lecture time in the respiratory infections lecture series to accommodate coverage of the novel SARS-CoV-2 in Fall 2020 by organizing content methodically with consistent themes and paradigm patterns. The MedMicroMaps system uses an arching orientation of cardinal directions north/south/west/east, starting with clinical presentation organized by onset, reading left-to-right/west-to-east for acute (less than 1 week) to chronic (greater than 3 weeks) (Fig. 1, MedMicroMaps compass). The coordinates of top-to-bottom/north-to-south are guides for anatomical location, corresponding to proximal to distal or superficial to deeper organs. The microbe biological classifications are arranged with smallest on top to the largest on the bottom, with viruses on top-left/north-west and helminths on bottom-right/south-east. Furthermore, the microbial colors correspond to staining methods when applicable with Gram-positive bacteria marked as Purple for crystal violet stain and Unicellular Fungi as Blue for lactophenol blue stain. The position of the microbes correlates to disease onset, representing that the microbes in the western quadrants (viruses, typical bacteria) mostly cause acute infections and the microbes in the eastern quadrants (atypical bacteria, fungi, parasites) mostly cause chronic infections.

The MedMicroMaps sub-sections are organized by organ system and each module starts with a disease presentation for that respective organ system. The key clinical findings align with the process of decision tree logic of clinical reasoning for physicians-in-training, frequently encountered with board-style multiple-choice questions with a clinical vignette [6]. The specific category of disease, ex., acute, upper respiratory infection, has an arrow hyperlink that routes a user to an epidemiological map, listing causative agents of respiratory infections, ranked by severity or incidence, and further clustered by epidemiological factors, ex., elderly versus pediatric, exposure risk, occupation, or medical history (see Supplemental Material, Respiratory MedMicroMaps). The next feature of MedMicroMaps module links the user to the clinical microbiology diagnostic algorithms arranged by biological classifications, based on common patterns for *e*-learning resources [7]. The Microbe Biology maps use consistent legends with color-coding microbes and pattern organization, ex., DNA on top and RNA on bottom for viruses, Cocci on top and Bacilli on bottom for typical bacteria, Unicellular on top and Multicellular on bottom for fungi and parasites. Lastly, each organ system map has a complete Overview slide to provide consistent outlines for all diseases and a broad guide of the range of infectious agents for a system. The hyperlinks allow the user to toggle back and forth to consider differential diagnoses to include or exclude microbes based on clues provided in a clinical scenario with follow-up laboratory findings.

## Results

**Fig. 2:**
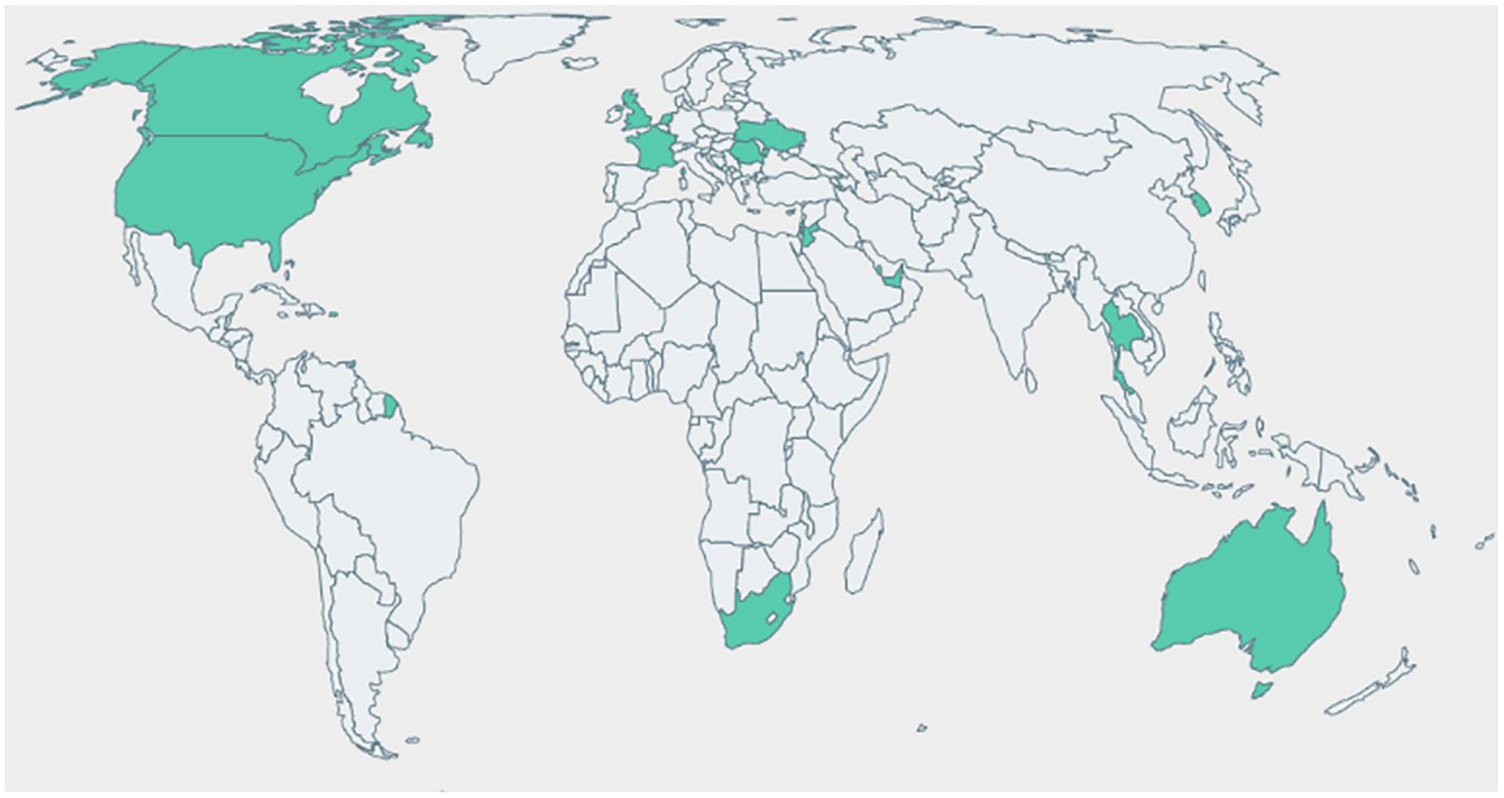
User engagement assessed with QR code scans for Microbiology Digital Media Resources website. Supplemental* e*-learning MedMicroMaps resources were provided on the Digication website server, with subpages for organ system modules. The resource was announced for second-year medical students (*n* = 865 during hybrid synchronous lecture in March 2022 with a QR code provided in lecture PowerPoint. Website viewing on the day of announcement was tracked with QR Tiger scans and follow-up website views were determined with monthly summaries from Digication with estimates represented as 1 K = 1000, with 16 K views acquired over 14 months

**Fig. 3:**
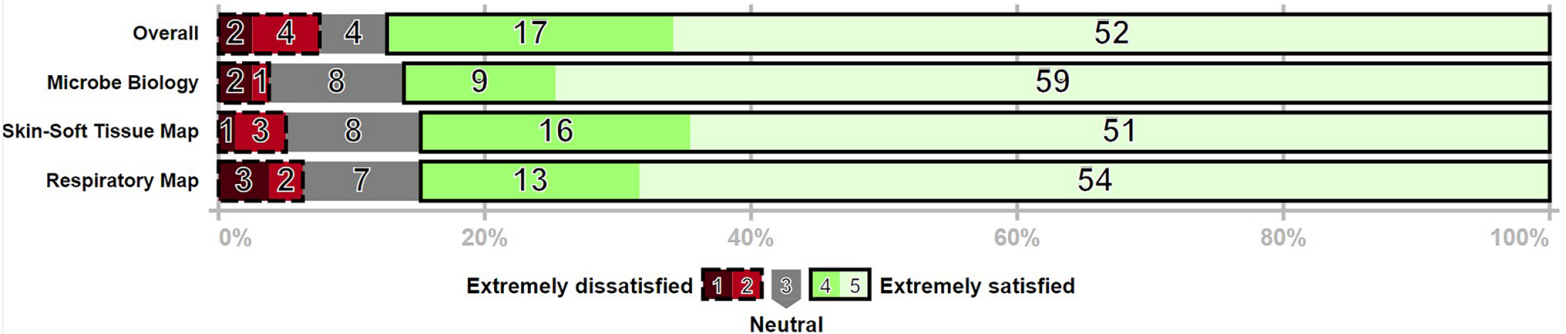
Student feedback survey on digital media resources. A link to the Qualtrics Student Feedback survey was provided via email announcement on Sakai LMS after the last infectious disease module. A 5-point Likert scale was used for student responses (*n* = 79) to prompts: “Rate your level of satisfaction with the Microbiology digital study resources (Overall, Microbial Biology Classification MedMicroMaps, Skin/Soft Tissue Infections MedMicroMaps and Respiratory Infections MedMicroMaps)”

Students in term 4 Spring 2022 (*n* = 865) at St. George’s University, School of Medicine on the Caribbean Island of Grenada in hybrid delivery format were provided a link to the Microbiology Digital Media Resources website hosted on the Digication web server via QR code announcement during the first synchronous lecture of infectious disease system module. An office hour session delivery via Zoom was offered in the Respiratory module, which consisted of Case-Based Guide to the MedMicroMaps to integrate the decision tree logic through a case series. The office hour recording was posted to Sakai LMS. A link to Feedback Survey in Qualtrics with IRB-approval was provided to students enrolled in term 5 via course email announcement on the LMS Sakai server. Results of single-day engagement showed 498 QR code scans, tracked with QR Tiger, with global distribution of scans covering 4 continents and 16 countries (Fig. 2, user engagement QR scans). User views on the website indicated 1000 + views per module per month across 2 cohorts over 10 months, with increased viewing the weekend spiking prior to the module exam. A limitation of the study was encountered with viewer statistics reported as 2 K, 3 K, etc. without daily count granularity. After the final infectious disease lecture in term 5, 79 students (9.1% response rate) completed the Qualtrics survey (Fig. 3, student feedback). Most of the responses indicated Extremely Satisfied to “Rate your satisfaction with the Microbiology digital study resources” with the highest rating for Microbe Biology MedMicroMaps (75%, *n* = 59), followed by Respiratory MedMicroMaps (68%, *n* = 54), Overall Resources (66%, *n* = 52), and lastly Skin and Soft Tissue Infections (65%, *n* = 51). When prompted for specific utilization of the *MedMicroMaps*, students ranked Exam Preparation most frequently (63.2%, *n* = 50), followed by used with Practice Questions (54.4%, *n* = 43). User engagement of Case-Based Guide to MedMicroMaps indicated a higher usage of Zoom-recorded sessions the weekend after posting (44.3%, *n* = 35) as compared to attending live Zoom sessions (34.1%, *n* = 27) (data not shown).

## Discussion

The findings of the pilot study support utilization of MedMicroMaps as a supplemental *e*-learning tool for medical students. The early development and delivery of the resource relied on posting individual system PowerPoint files in the Sakai LMS in subfolders based on lecture dates, starting with Microbe Biology in term 3 and Skin and Soft Tissue in term 4. The Digication website was created to host the comprehensive collection of system-based MedMicroMaps with extra digital media resources, including animations, office hour recordings, and fill-in-the-blank study guides. The data from the QR code tracking (Fig. 2) validates the scalability of *e*-learning resources for global audiences. Challenges encountered during the pilot study were lack of familiarity for the resource availability and lack of understanding how to integrate the system with practice questions or exam study. As the students progressed through the systems in terms 4 and 5, the author of the study reinforced the decision tree logic with the MedMicroMaps-embedded hyperlinks in the Respiratory Infections module lectures, which greatly increased awareness of the resource and engagement with the Digication website for future system modules in terms 4 and 5. The project is ongoing at the Montana College of Osteopathic Medicine, a new branch campus of Rocky Vista University, with curriculum design for all infectious disease content based on MedMicroMaps organization by organ system and microbial classification. To expand broader student access, the digital media resources have been moved to the Google domain website www.medmicromaps.com as the Digication server restricts to institutional login. A longitudinal study is warranted to determine the impact of MedMicroMaps on learning outcomes for short-term recall for module exams and for long-term recall for board exam preparation.

The MedMicroMaps system was created with hyperlinking features on 2-D PowerPoint format and was designed with expansion to cross-platform digital media interfaces, which is an expanding field in biomedical education [8]. The format of algorithm decision trees is compatible with a large language model in machine learning which can be utilized to develop novel immersive learning formats including virtual reality (VR), augmented reality (AR), metaverse (collectively referred as XR Extended Reality), *e*-book, and mobile/tablet app with course-tailored study guides and clinical cases. Digital representations of microbes can be accurately modeled by size and shape, utilizing computational power of artificial intelligence to generate a future microbial world in immersive learning platforms. MedMicroMaps in cross-platform format will be expanded for story-line experiences of all infectious diseases and can be adapted for utilization for diverse audiences within the field of biomedical education including allopathic and osteopathic medicine, nursing, pharmacy, and veterinary sciences and can be further modified to academic levels from undergraduate studies to practicing clinicians.

### Supplementary Information

Below is the link to the electronic supplementary material.

Supplementary file1 (PDF 397 KB)

## Data Availability

The author confirms that the data supporting the findings of this study are available within the article and its supplementary materials with original data sets from institutional servers (Qualtrics, Sakai, Panopto) accessible upon request.
