# Supplementary material for: A Mixed Methods Pilot Study to Evaluate User Engagement with MedMicroMaps: A Novel Interactive E-learning Tool for Medical Microbiology
Source: Med Sci Educ. 2024 May 1;34(4):753–7. doi: 10.1007/s40670-024-02047-3 (PMC11296994; doi:10.1007/s40670-024-02047-3)
Supplement: Supplementary file 1 — Supplementary file1 (PDF 397 KB) [file 40670_2024_2047_MOESM1_ESM.pdf]

Respiratory MedMicroMap

Respiratory Infections

Acute Presentation

URTI
- Rhino-Pharyngitis
- Pharyngo-Conjunctivitis
- Sinusitis, Otitis Media, Epiglottitis
- Mumps
- Pharyngitis, Tonsillitis

LRTI
- Bronchitis
  - Whooping Cough
  - Croup
  - Bronchitis Bronchiolitis
- Viral Pneumonia
  - Flu
  - COVID-19
- Bacterial Pneumonia
  - Bacterial Typical Pneumonia
  - Bacterial Atypical Pneumonia

Bioterrorism
- Inhalational anthrax
- Plague
- Hanta pulmonary syndrome

Chronic Presentation

URTI
- Sinusitis, Otitis Media
- Tonsillitis, Peritonsillar Abscess

LRTI
- Tuberculosis
- Fungal infections
- Q Fever
- Melioidosis

Superficial or Proximal Anatomy

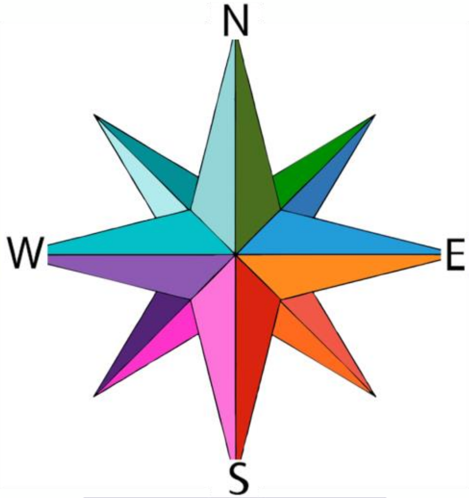

Acute Onset

Chronic Onset

Deeper or Distal Anatomy

MedMicroMaps– Dr. Jane Harrington @2021

Acute URTI MedMicroMap

Acute URTI

- **Rhino-Pharyngitis**
  - Rhinovirus — All ages
  - Common Coronavirus — All ages
  - Epstein-Barr, CMV — Young adults
  - Measles, Rubella — Unvaccinated
- **Pharyngo-Conjunctivitis**
  - Adenovirus — Crowded conditions (schools, military), swimming pools,
- **Sinusitis, Otitis Media, Epiglottitis**
  - *S. pneumoniae* — All ages, — Pediatric <10 years Unvaccinated (Hib)
  - *Haemophilus influenzae* — All ages — Pediatric <10 years Unvaccinated (Hib)
  - *Moraxella catarrahlis* — All ages — Pediatric <10 years
- **Mumps**
  - Mumps — Crowded conditions — Unvaccinated
- **Pharyngitis, Tonsillitis**
  - *S. pyogenes* — Pediatric < 10 years
  - *C. diphtheriae* — Endemic travel — Unvaccinated

Virus (DNA, Env)
Virus (RNA, Naked)
Gram Positive
Gram Negative
Atypical Bacteria
Fungus
Parasite

HIGHEST EXPOSURE RISK OR HIGHEST INCIDENCE	HIGHEST Risk of Complications

MedMicroMaps– Dr. Jane Harrington @2021

Acute LRTI MedMicroMap

Acute < LRTI

- **Bronchitis**
  - Whooping cough → *Bordetella pertussis* — All ages — Pediatric < 4 years unvaccinated
  - Croup → Parainfluenza PIV — Pediatric < 10yrs
  - Bronchitis bronchiolitis → Respiratory Syncytial Virus RSV — Pediatric < 2 yrs
- **Viral pneumonia**
  - Flu → Influenza — All ages — > 60 years, unvaccinated
  - COVID-19 → SARS-CoV2 — All ages — >60 yrs, Hypertension, Diabetes, Obesity
  - ARDS
    - → Adenovirus — Military recruits
    - → Respiratory Syncytial Virus RSV — Pediatric
- **Bacteria pneumonia**
  - Bacterial typical pneumonia
    - → *S. pneumoniae* — All ages — COPD/CF, asplenic
    - → *S. aureus* — Preceding flu
    - → *Haemophilus influenzae* — Kids < 10 yr — COPD/CF
    - → *Moraxella catarrahlis* — Kids < 10 yr — COPD/CF
    - → *Klebsiella pneumoniae* — Hospitalized — Alcoholics
    - → *Bacteroides* GNAR — Hospitalized — Alcoholics
    - → *Pseudomonas* — Hospitalized — COPD/CF
  - Bacterial atypical pneumonia
    - → *Mycoplasma* — Adolescent, elderly
    - → *Chlamydia* spp. — Adolescent, birds (*C. psittaci*)
    - → *Legionella* — Smokers, aerosolized water

Pediatric

Elderly

HIGHEST EXPOSURE RISK OR HIGHEST INCIDENCE | HIGHEST Risk of Complications

Legend: Virus (DNA, Env) · Virus (RNA, Naked) · Gram Positive · Gram Negative · Atypical Bacteria · Fungus · Parasite

Acute Bioterrorism MedMicroMap

Acute < Bioterrorism

- Inhalational anthrax → *Bacillus anthracis* | Inhalation of spores
- Plague → *Yersinia pestis* | Fleas or infected human
- Tularemia → *Francisella tularemia* | Ticks, infected rabbits
- Hanta pulmonary syndrome → Hantavirus | Inhalation of rodent urine

Virus (DNA, Env)
Virus (RNA, Naked)
Gram Positive
Gram Negative
Atypical Bacteria
Fungus
Parasite

HIGHEST EXPOSURE RISK OR HIGHEST INCIDENCE | HIGHEST Risk of Complications

MedMicroMaps– Dr. Jane Harrington @2021

Chronic URTI MedMicroMap

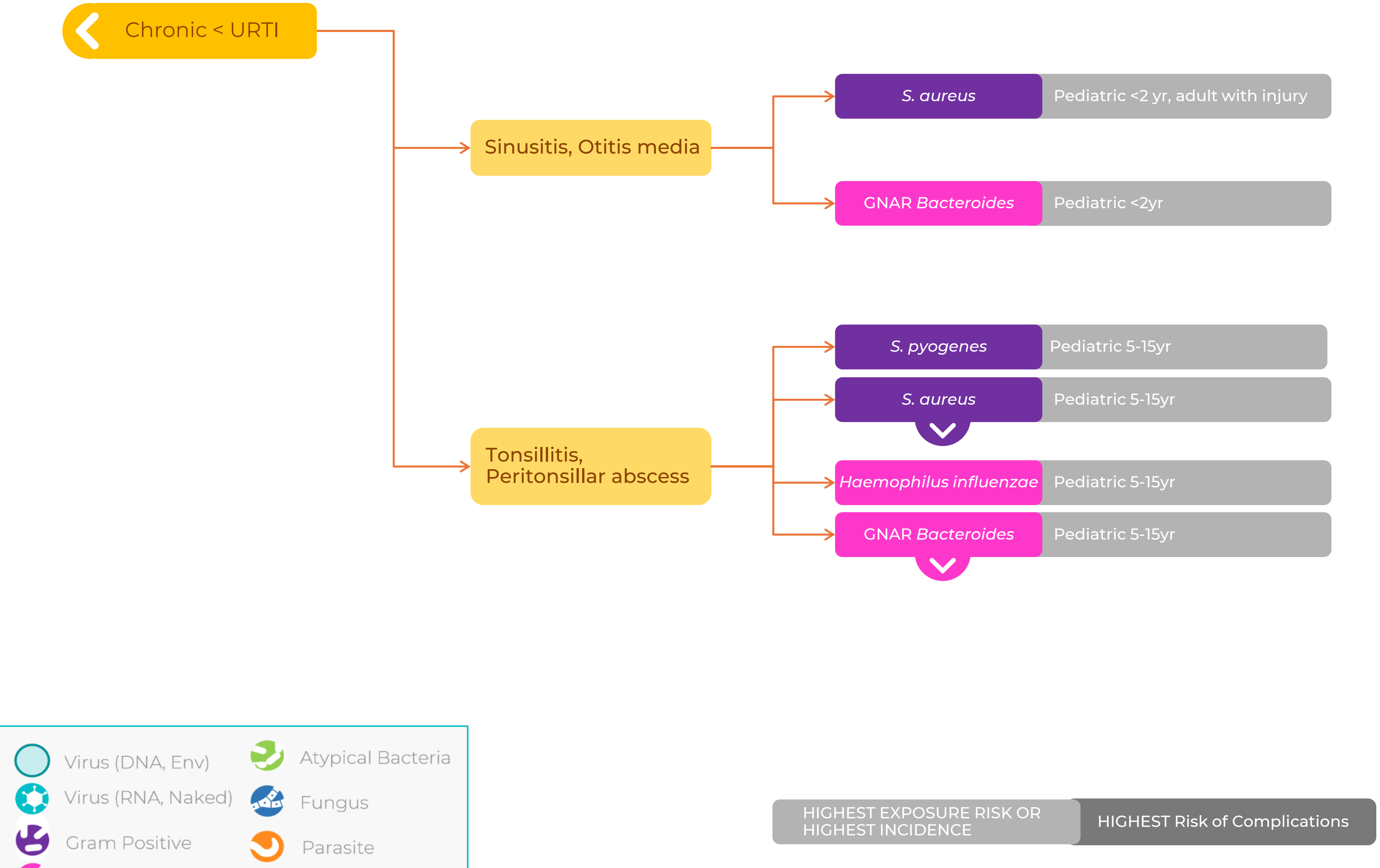

Chronic LRTI MedMicroMap

Chronic < LRTI

- **Tuberculosis**
  - *Mycobacterium tuberculosis* — Endemic travel — Low CMI CD4+ T cells
  - *Nocardia asteroides* — Soil inhalation — Low CMI CD4+ T cells
- **Fungal infections**
  - *Histoplasma* — Caves, bats/bird droppings
  - *Coccidioides* — SW USA, desert
  - *Blastomyces* — Ohio River Valley, soil
  - *Cryptococcus* — Bird droppings (pigeons)
  - *Aspergillus* — Neutropenia, construction
  - *Pneumocystis jirovecii* — HIV-AIDS <200 T cell count
- **Q Fever**
  - *Coxiella burnetti* — Domestic livestock
- **Melioidosis**
  - *Burkolderia pseudomallei* — SE Asia, Austraila

Virus (DNA, Env)
Virus (RNA, Naked)
Gram Positive
Gram Negative
Atypical Bacteria
Fungus
Parasite

HIGHEST EXPOSURE RISK OR HIGHEST INCIDENCE | HIGHEST Risk of Complications

MedMicroMaps– Dr. Jane Harrington @2021

Respiratory Infections Virus MedMicroMap

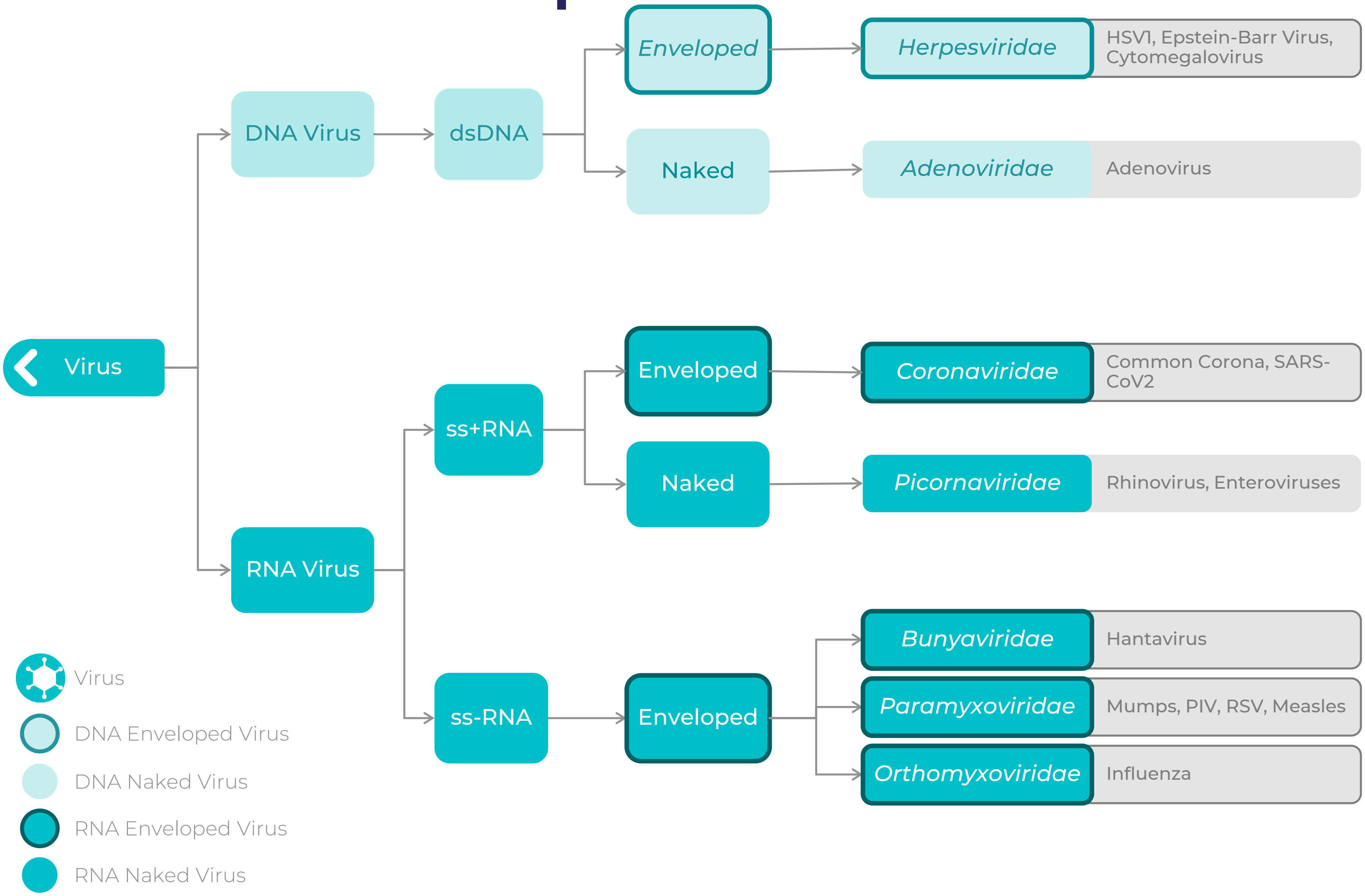

MedMicroMaps– Dr. Jane Harrington @2021

Respiratory Infections
Gram Positive Bacteria MedMicroMap

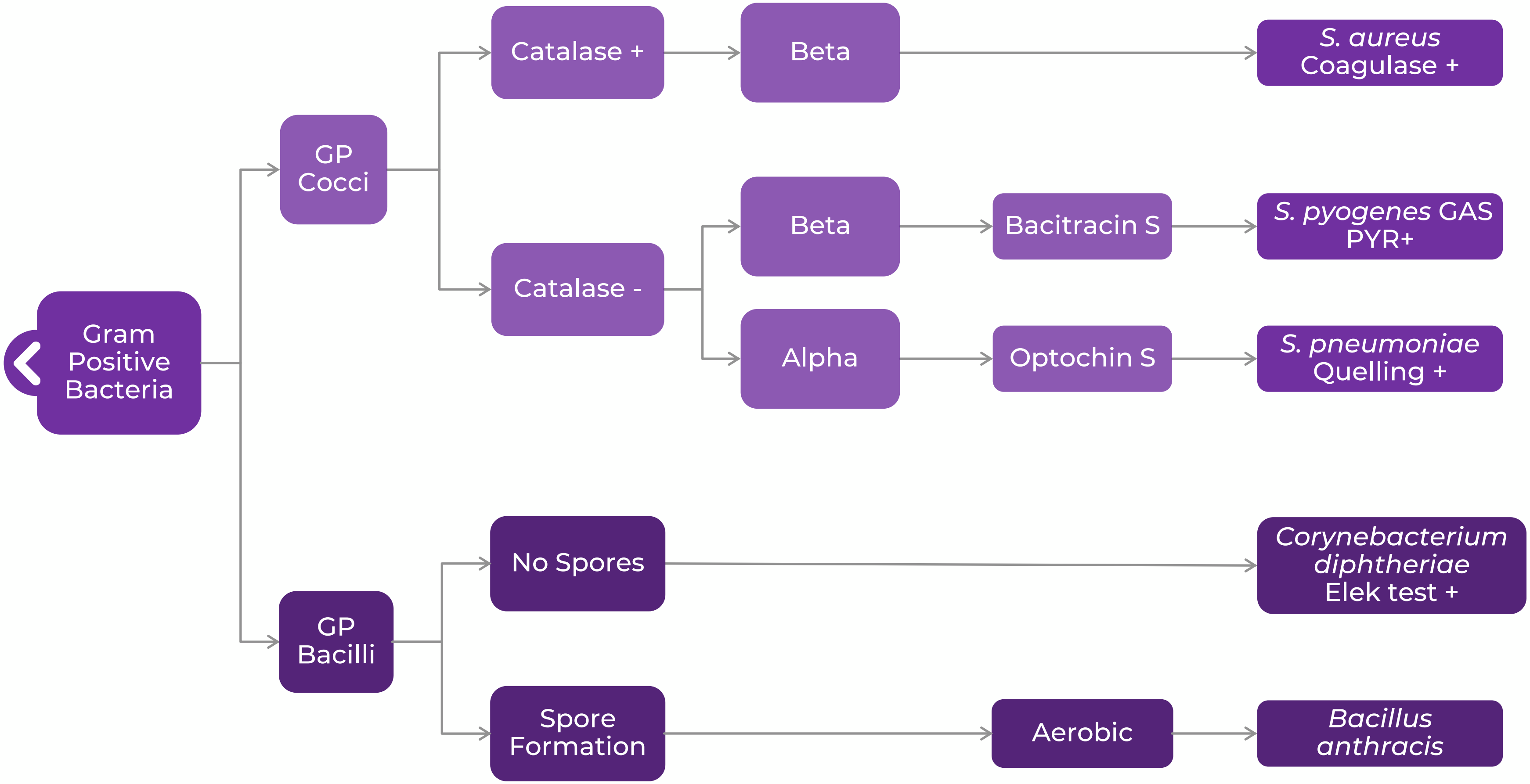

Gram Positive

MedMicroMaps– Dr. Jane Harrington @2021

Respiratory Infections
Gram Negative Bacteria MedMicroMap

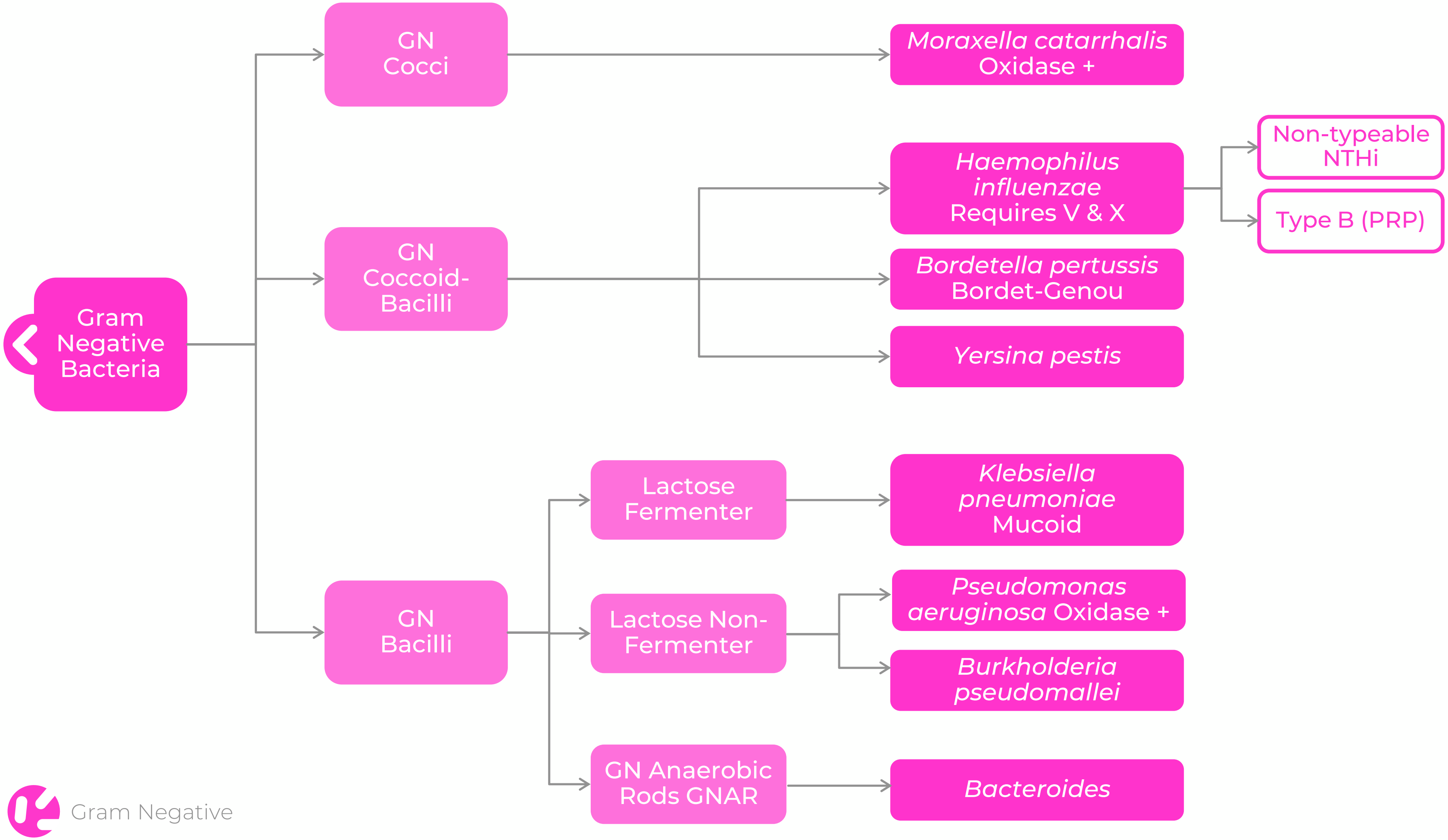

Gram Negative

MedMicroMaps– Dr. Jane Harrington @2021

Respiratory Infections
Atypical Bacteria MedMicroMap

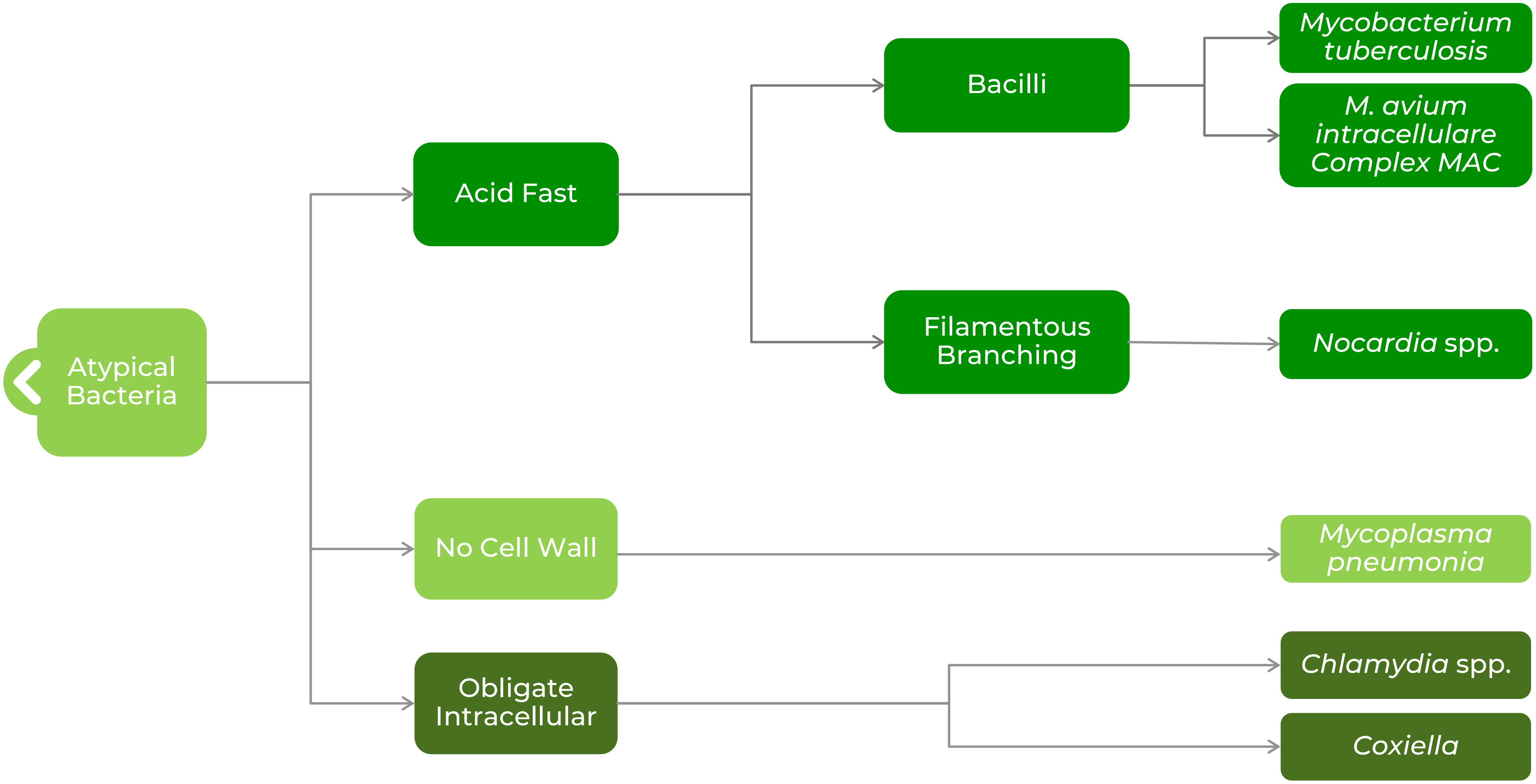

Atypical Bacteria

MedMicroMaps– Dr. Jane Harrington @2021

Respiratory Infections
Fungus MedMicroMap

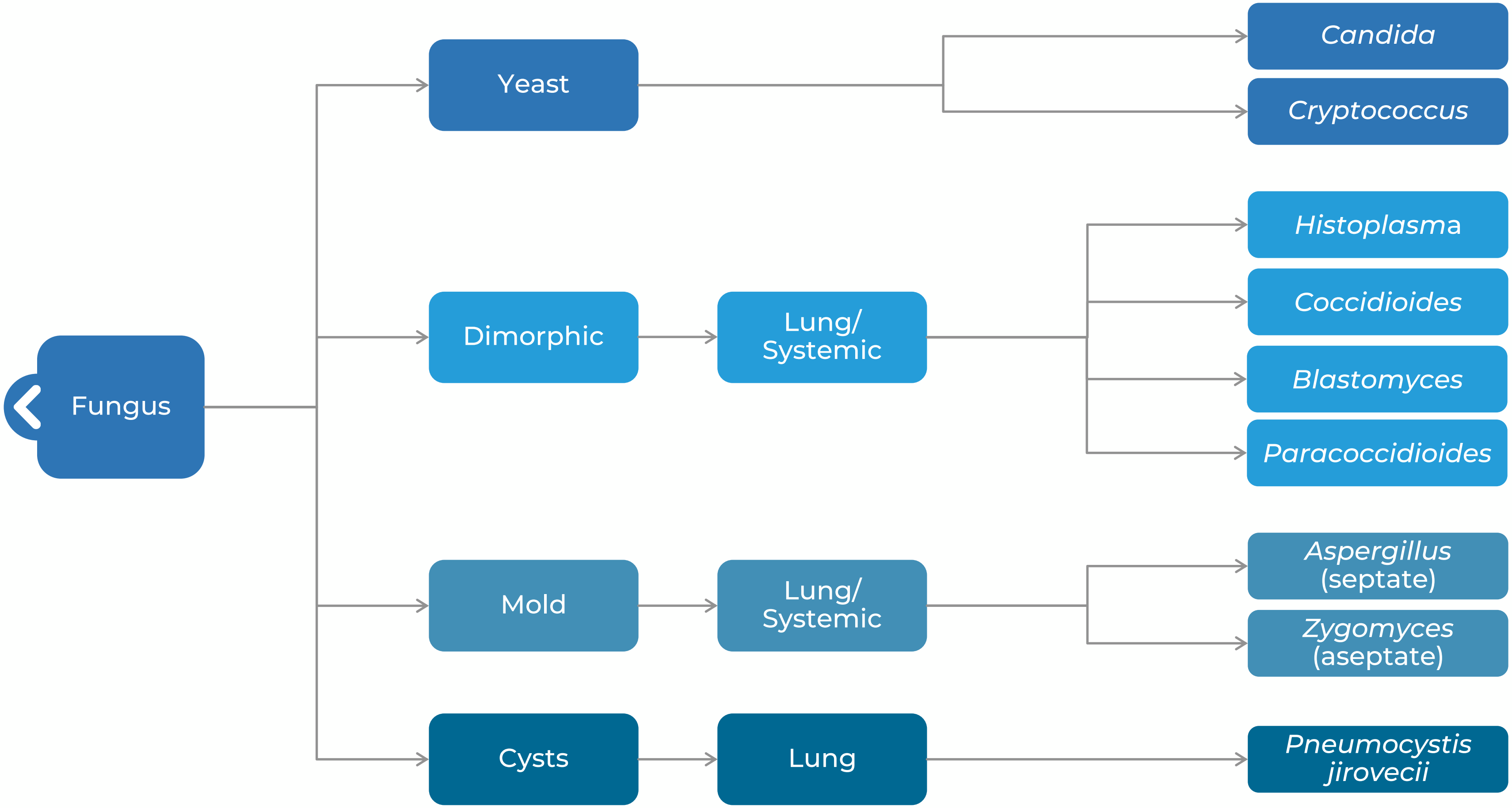

Fungus

MedMicroMaps– Dr. Jane Harrington @2021

Respiratory MedMicroMap Overview

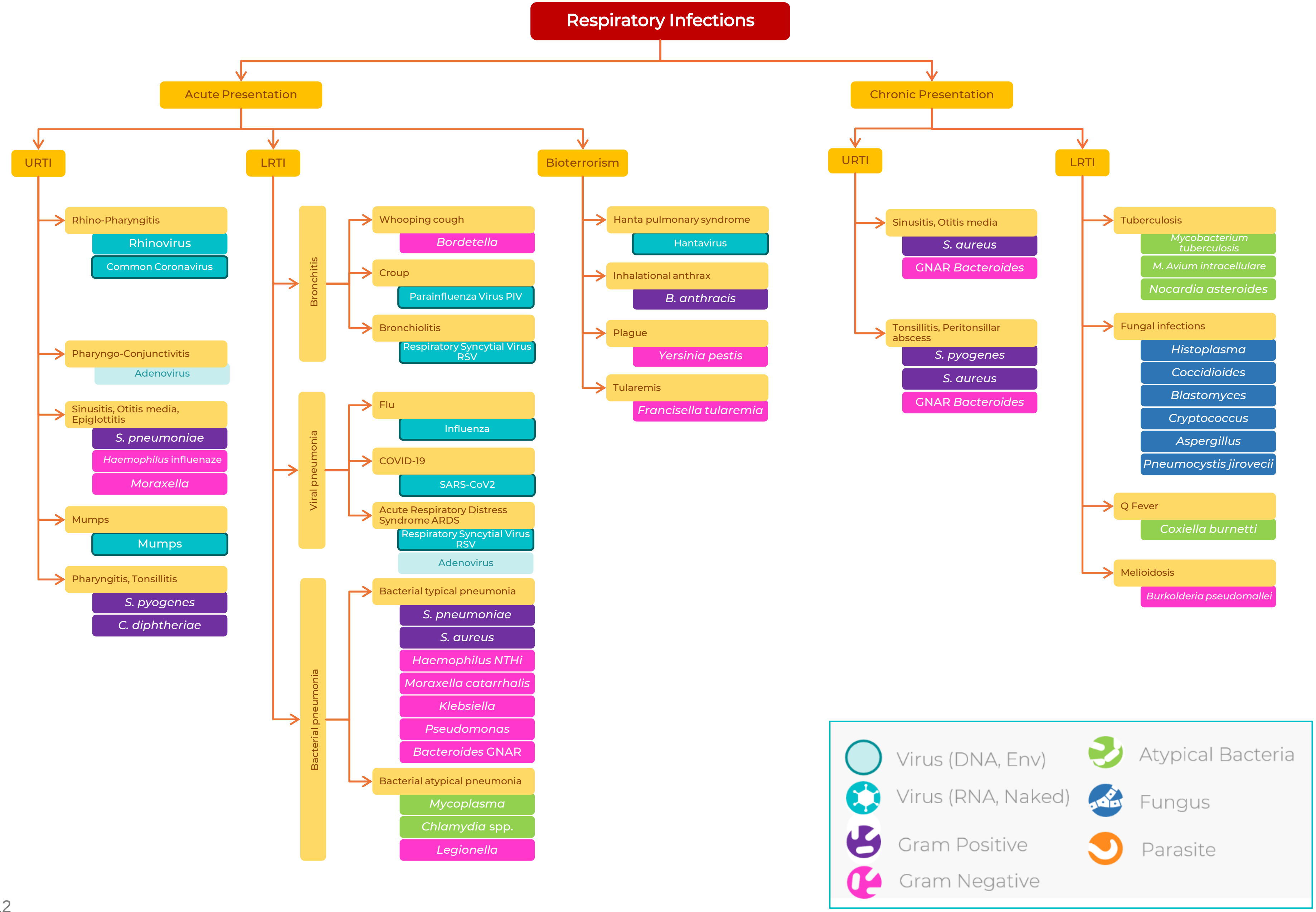

MedMicroMaps– Dr. Jane Harrington @2021
